# Nutritional Management of Patients with Head and Neck Cancer—A Comprehensive Review

**DOI:** 10.3390/nu15081864

**Published:** 2023-04-13

**Authors:** Dinko Martinovic, Daria Tokic, Ema Puizina Mladinic, Mislav Usljebrka, Sanja Kadic, Antonella Lesin, Marino Vilovic, Slaven Lupi-Ferandin, Sasa Ercegovic, Marko Kumric, Josipa Bukic, Josko Bozic

**Affiliations:** 1 Department of Maxillofacial Surgery, University Hospital of Split, 21000 Split, Croatia; 2 Department of Anesthesiology and Intensive Care, University Hospital of Split, 21000 Split, Croatia; 3 Department of Pathophysiology, University of Split School of Medicine, 21000 Split, Croatia; 4 Department of Pharmacy, University of Split School of Medicine, 21000 Split, Croatia

**Keywords:** head and neck surgery, oromaxillofacial surgery, head and neck cancer, clinical nutrition

## Abstract

While surgical therapy for head and neck cancer (HNC) is showing improvement with the advancement of reconstruction techniques, the focus in these patients should also be shifting to supportive pre and aftercare. Due to the highly sensitive and anatomically complex region, these patients tend to exhibit malnutrition, which has a substantial impact on their recovery and quality of life. The complications and symptoms of both the disease and the therapy usually make these patients unable to orally intake food, hence, a strategy should be prepared for their nutritional management. Even though there are several possible nutritional modalities that can be administrated, these patients commonly have a functional gastrointestinal tract, and enteral nutrition is indicated over the parenteral option. However, after extensive research of the available literature, it seems that there is a limited number of studies that focus on this important issue. Furthermore, there are no recommendations or guidelines regarding the nutritional management of HNC patients, pre- or post-operatively. Henceforth, this narrative review summarizes the nutritional challenges and management modalities in this particular group of patients. Nonetheless, this issue should be addressed in future studies and an algorithm should be established for better nutritional care of these patients.

## 1. Introduction

Head and neck cancers (HNC) represent epithelial malignancies in the paranasal sinuses, nasal cavity, oral cavity, pharynx, and larynx, all of which significantly impact the morbidity and mortality of the affected population [1]. Hence, the National Cancer Institute’s (NCI’s) Surveillance, Epidemiology, and End Results (SEERs) refer to more than 65,000 new cases annually, with more than 14,500 death cases associated with the disease [2].

Most cases of HNC are head and neck squamous cell carcinoma (HNSCC), which is the sixth most widespread malignancy worldwide with an estimated 3% of all cancer cases [3]. The most significant risk factors that contribute to its pathogenesis are tobacco usage, alcohol consumption, and infection with a high-risk human papilloma virus [4]. Furthermore, the most lethal among the HNSCC is the oral squamous cell carcinoma (OSCC), which accounts for 90% of all oral malignancies and has an estimated 2–3% death rate of all cancer-related deaths [5].

Even though there are several different treatment modalities for HNC, due to the complex anatomy along with the highly important functions in the head and neck region, management of HNC should be multidisciplinary, with the focus not only on therapy but also on supportive pre and aftercare [6]. One of the significant reasons for the high HNC disease burden is malnutrition and nutritional deficits in these patients, which have a substantial impact on health outcomes as well as the overall quality of life [7]. Malnutrition can be referred to by following several different criteria, including more than 5% weight loss in three months or more than 10% in a six-month period, or a BMI less than 20 kg/m^2^, while albumin levels less than 35 g/L can be suggestive of malnutrition as well [8,9,10].

Problems with nutrition already start with disease onset, with various studies showing 25–65% of HNC patients presenting themselves with malnutrition, with more than 10% weight loss from the normal body mass [11,12,13]. High levels of variability and difficulties to obtain exact data are probably driven by different malnutrition definitions and different methods of malnutrition assessment [14]. However, there could be several reasons for the still significant percentages of malnutrition in these patients even before treatment, including chronic malnutrition, which is associated with alcohol and tobacco usage, trismus, obstruction of the respiratory and digestive system with aspiration, odynophagia, and dysphagia [6,13]. Hence, Kubrak et al. showed in a large cohort of HNC patients that independent predictors of weight loss in a naive population were tumor stage, dietary intake categories, and performance status [15].

Different treatment modalities can only further worsen the nutritional status of HNC patients. These modalities include chemotherapy, radiation therapy, surgical procedures, specialized targeted therapies, and different combination therapies [16]. While surgical procedures are a potential cause of disrupted food intake, mostly based on tumor location and resection type and size, radiotherapy, and chemotherapy are connected to various symptoms that further impair oral food intake and often cause treatment withdrawal [17,18]. Furthermore, according to several studies, malnutrition in HNC patients during therapy can be present in substantially high percentages, up to 80% [19,20,21]. Mucositis is commonly associated with chemotherapy and radiotherapy and is characterized by inflamed lesions in the area of the mouth and throat. These complications are connected to infections, dysphagia, and pain, among others [22]. Xerostomia and impaired parathyroid gland function are two of the most common adverse effects of radiotherapy that significantly reduce the quality of life of patients and are often associated with thick saliva. This causes further negative effects on chewing, swallowing, and speaking, with an accompanying increase in infections [23,24]. Other adverse effects of therapeutic modalities that significantly impair weight maintenance and the proper healing process include nausea, vomiting, constipation, depression, dysgeusia, and odynophagia [6].

Adequate nutritional support in HNC patients is of vital importance, as malnutrition is associated with a decreased therapeutic and immunological response with a higher incidence of infections and post-surgical complications. Furthermore, it causes treatment breaks with a higher economic burden and decreased functional performances that cumulatively lead to decreased quality of life and higher mortality rates. Interestingly, overweight and obese people with higher BMIs are at the same risk of all of the mentioned complications [6,13,25,26,27]. Hence, studies have shown that weight loss before treatment was one of the main independent survival predictors, while nutritional support before surgery can lead to significant beneficial changes in quality of life with fewer post-operative infectious complications [28,29].

These considerations were leading to the development of various screening tools in order to diagnose vulnerable patients and to prevent treatment complications and negative health outcomes. Moreover, the Nutritional Risk Screening 2002 (NRS 2002) and Malnutrition Universal Screening Tool (MUST) were accepted by the European Society for Clinical Nutrition and Metabolism (ESPEN) for hospital usage [19]. The newest edition of the ESPEN clinical guidelines for clinical nutrition in cancer, which is dedicated to all healthcare professionals that participate in cancer patient care, has a total of 43 recommendations and short commentaries regarding cancer patient management. Even though general recommendations are included that can be of use in HNC patient care, with some specialized parts that address this population specifically, there is still a need for comprehensive, detailed guidelines that involve only the HNC patient population [30].

Another important practice that should be implemented regularly in HNC patient management is involving enhanced recovery after surgery (ERAS) protocols. These specialized, evidence-based protocols were initially presented in the perioperative care of colorectal surgery, and up to today investigations have shown numerous beneficial health outcomes with managed patients in different surgical branches [31,32]. The ERAS protocol with published recommendations was introduced for HNC surgical procedures with free flap reconstruction in 2017, with a special focus on perioperative nutritional care [33]. According to these recommendations, Moore et al. performed a clinical investigation on 25 HNC surgery patients that received perioperative nutritional supplementation. Results have shown that processes still need some modifications to ensure specialized approaches for patient care improvement [34].

These data suggest that even though some nutritional guidelines for HNC surgery exist, there is still a potential need for new investigations, modifications, and better clinical utilizations.

## 2. Nutritional Modalities

Patients with head and neck cancer, due to the location of the tumor in the upper airway and digestive tract, can have difficulties masticating and swallowing food. Tumor-related dysphagia can significantly compromise the nutrition status of those patients and put them at risk of malnutrition at the time of diagnosis. Due to inadequate nutrition intake, these patients are prone to weight loss, decrease in muscle mass, fatigue, and anemia, which ultimately leads to cancer cachexia syndrome. Studies indicate that impaired nutrition status is noticed in 25–27% of these patients at the time of diagnosis and before the start of treatment [35,36,37,38]. It was also shown that pretreatment weight loss is a strong survival predictor and pretreatment cachexia is connected to poor survival [29,38,39]. Additionally, sarcopenia that occurs as a result of cancer cachexia has been connected with unfavorable treatment outcomes in patients with head and neck cancer [40]. Cancer treatment can further aggravate treatment-related dysphagia, resulting in reduced food intake and deteriorating nutrition status [35,36,37]. Severe weight loss (more than 10% of body weight) during treatment has been observed in the absence of intensive nutrition support in up to 58% of patients [38,41,42].

Nutrition support is a crucial part of head and neck cancer control, supporting better disease outcomes. Oral intake of food is the preferred method of nutrition, but in cases when adequate nutritional intake cannot be maintained by mouth, enteral or parenteral nutrition is necessary. Enteral nutrition is preferred over parenteral nutrition, being physiologically natural. It also maintains gastrointestinal integrity, protecting it from atrophy, and also supports gut immune function (Table 1). Enteral nutrition implies the administration of food into the gastrointestinal tract through a tube or stoma.

Furthermore, it is important to plan the nutritional management of these patients during pre-treatment care. Although head and neck cancer patients have restricted oral intake, their gastrointestinal tract is usually functional. Therefore, enteral nutrition is indicated in patients that are unable to feed orally but have a functioning and accessible gastrointestinal tract. It can be short-termed or long-termed and can be administrated into the stomach—gastric or into the intestine—post-pyloric [43]. It is a safe and effective nutrition manner to provide nutrition that can be easily administered by patients and their families at home. The main contraindication for applying enteral nutrition is the non-functional gastrointestinal (GI) tract. Numerous studies have shown that administrating feeding tubes before the start of treatment in these patients ensures better overall outcomes. It acts beneficially in the prevention of weight loss and sarcopenia, dehydration, reduced hospital admission, and improved quality of life [44,45,46,47,48,49]. Improvements in nutrition parameters, including anthropometrics and laboratory data, have also been observed [50].

## 3. Enteral Nutrition

Several factors must be considered in determining which type and modality of enteral nutrition are administrated. The site of feeding and expected duration is primary, but it is also important to consider timing and rate of initiation, feeding modality, risk of complications, and possible contraindications. An individual approach and careful assessment are key in ensuring clinically appropriate and nutritionally complete enteral nutrition. The following options are available: nasogastric tube, nasojejunal tube, gastrostomy, and jejunostomy (Table 2) [51].

### 3.1. Nasogastric Tube

A nasogastric tube (NGT) is a plastic catheter inserted through the nose, passing the oropharynx and esophagus to the stomach [52]. It is the most commonly used type of enteral nutrition (EN) in HNC patients with a functional gastrointestinal tract if the tumor is obstructive, thereby impacting swallow function [53]. Generally, NGTs are used for a shorter period (<4 weeks) and can be administered perioperatively (prophylactic) or postoperatively (reactive) [54].

Prophylactic enteral feeding is used when nutrition support is anticipated for an extended time after more invasive surgical procedures and in severely malnourished patients. The National Comprehensive Cancer Network (NCCN) published indications for prophylactic tube placement, summarized in Table 1 [55].

Nutritional status seems to be maintained or enhanced with tube implantation, according to Langius et al.’s systematic study, which found that prophylactic tube feeding increases nutritional intake and nutritional status compared to oral consumption alone [56].

If oral intake is not possible after 4–6 weeks, percutaneous endoscopic gastrostomy (PEG) is indicated [54]. The literature showed similar nutritional and clinical outcomes in patients with NGTs and PEGs. However, complications such as increased tube dislodgement and chest infection were noticed after a long period in patients with NGTs. Additionally, compared to patients with gastrostomies, patients with nasogastric tubes reported greater body image problems, difficulty with feeding, and considerable social activity interruption [57,58].

Still, NGTs remain a routine modality of enteral nutrition because there are no notable differences in overall complication rates. They are easily placed, significantly less expensive, and the transition from enteral nutrition back to oral nutrition is shorter than PEG tubes [59].

### 3.2. Nasojejunal Tube

Oroenteric or nasoenteric feeding tubes are intended for enteral nutrition into small intestine regions, meaning the duodenum and jejunum. Application of nutrients in the small intestine is indicated for short-term feeding in patients that have impaired gastric motility since they are at risk for gastric emptying delay, reflux, and aspiration. It is also instructed to avoid gastric feeding in patients with severe acute pancreatitis and promote enteral nutrition [60].

Enteric feeding tubes can be placed blindly at the bedside without the use of any technology. When placing the tube, the distal end must surpass the duodenojejunal flexure, otherwise, it can inevitably retract back to the stomach. Nasojejunal feeding tubes can be of various sizes. Usually, they are small-bore with flexible tips that are intended to ensure spontaneous passage into the small intestine and protect from injury of mucosa or perforation. The tubes are also provided with stylets or guide wires providing structure for easier placement [61,62]. Studies suggest that blinded positioning of a small-bore feeding tube by a well-trained and experienced clinician has a success rate of 80% or more [63].

Possible complications of the application of nasojejunal tube and nasojejunal tube feeding are epistaxis, sinusitis, oesophageal ulceration or strictures, blockage, malposition or injury, and perforation of the gastrointestinal (GI) tract. Malposition implies placing the tube outside the GI tract. Placing it in the bronchopulmonary tree can cause infection, effusion, or empyema [51,63,64,65,66]. As a rare occurrence, but a very severe complication, it can malposition intracranially. To reduce the risk of malposition or injury, tubes can be placed using endoscopy or fluoroscopy, or magnetic guidance. Several studies show that using technology for placement increases success rates by 90% [66,67,68,69]. In addition, it is important to verify the proper position of the tube after placement using an abdominal X-ray.

There are indications that jejunal feeding may assist in the increased delivery of nutrients since there are fewer interruptions compared to gastric feeding, which is often interrupted by the application of medication and lavage [70,71]. It is propounded that post-pyloric tubes are more comfortable for patients than gastric tubes, so they are a good alternative for patients that show discomfort with NGT, decreasing removal of the tube by the patients [72]. Preferred modality of post-pyloric feeding should be continuous to avoid discomfort and dumping syndrome that occurs with bolus feeding. However, for HNC patients, a NGT is still the preferred option over the nasojejunal tubes, and the latter are usually only reserved for patients who have impaired gastric motility.

### 3.3. Percutaneous Endoscopic Gastrostomy

Percutaneous endoscopic gastrostomy (PEG) is nowadays considered the “golden standard” for feeding and nutrition support in oral/maxillofacial surgery patients undergoing extensive surgery treatments. PEG tube placement is a well-accepted and frequently conducted procedure worldwide that has replaced surgical and radiological gastrostomy techniques [73]. Morbidity linked with PEG can vary from 5 to 10.3%, of which 3% are major adverse events [74].

The main indication for PEG in oral and maxillofacial patients is providing oral intake and meeting metabolic requirements due to the closeness of the cancer and organs that are in charge of normal food intake [6,75]. It is important to highlight that patients with maxillofacial region tumors are usually excessive smokers and/or alcohol consumers, with inadequate eating habits prior to diagnosis or treatment [76]. This group of patients often faces postoperative swallowing disorders, changed anatomy of the oral cavity due to surgery, impaired function of the tongue, dysphagia, odynophagia, dysgeusia, xerostomia, and nausea [6]. Malnutrition and weight loss are connected with poorer treatment outcomes, poor quality of life, and consequently elevated rates of morbidity and mortality [77].

The PEG can be permanent or temporary depending on the patient’s requirements. Since the nasoenteric tubes are usually used in patients with preserved airway reflexes who require enteral feeding for less than 30 days, PEG is currently the method of choice for medium to long-term enteral feeding [36]. It is important to emphasize the fact that preventively placed PEG tubes resulted in lower complication rates in comparison to therapeutically inserted PEGs [78].

Regarding the insertion techniques, there are three most commonly used methods: the “pull”, “push” (guide wire), and introducer (Russel) methods. All of them have the same concept of insertion through the abdominal wall at the spot where the abdominal wall and stomach are the closest. According to several studies, the success rate of PEG is 84–96% [79,80]. Since it could be unable to perform oral percutaneous gastrostomy for 4–7% of head and neck cancer patients, transnasal endoscopy is a method of choice. Oropharyngeal obstruction, severe trismus, or airway endangerment are the main causes of the transnasal approach.

Nasoenteric tubes contribute to a higher number of complications such as irritation, nasal decubitus, patient discomfort, ulceration, bleeding, esophageal reflux, and aspiration pneumonia [81]. They are also connected to poorer acceptance and psychological or social problems. Interestingly, compared to PEG, nasoenteric tubes have a lower feeding efficacy [82]. There is some strong evidence in the literature that the initiation of PEG feeding, as soon as the medical indication has been set up, can prevent further weight loss and contribute to patients’ quality of life.

The most common contraindications for the PEG placement are systemic, such as coagulation disorders, hemodynamic instability, or sepsis; disturbances at the site of placement in the abdomen, such as abdominal wall infection, ascites, peritonitis; and peritoneal carcinomatosis; and interposed organs such as the colon or liver [75,83].

The PEG tube insertion process is commonly considered harmless; however, some complications can take place. The mortality rate is expectedly higher in patients with underlying comorbidities [84]. Minor complications consist of wound infection, which is, according to the literature, somewhere between 5% and 25%. Since this probably occurs due to contamination by oral flora, antibiotic prophylaxis is recommended [85,86]. Granuloma formation is the next most common complication and is due to friction and excess moisture around the tube [87]. Moreover, some of the other possible minor complications are tube leakage into the abdominal cavity and consequent development of peritonitis; stoma leakage; tube obstruction; pneumoperitoneum; gastric outlet obstruction, and peritonitis [84,88].

Major complications are aspiration pneumonia; however, that is more often in neurologic patients due to feeding a large amount of content and being in the prone position than in OMFS patients; hemorrhage (retroperitoneal bleeding due to injuries of the gastric artery, splenic and mesenteric veins, and rectus sheath hematoma); necrotizing fasciitis, which is a potentially lethal complication; buried bumper syndrome (characterized by excessive tension amid internal and external bumpers that cause ischemia and necrosis of the gastric wall and consequently, the tube moves toward the abdominal wall), and perforation of the bowel [75,88,89,90].

Furthermore, it is important to highlight an interesting and unusual complication of PEG placement in HNC patients: the seeding of the tumor. It was observed that the seeding occurs during the “pull” or “push” method of PEG insertion when the tube collides with the tumor, directly transferring tumor cells [91]. Unfortunately, diagnosis is set when the tumor metastasis is large and visible. However, questions regarding this complication are still emerging and some studies imply that the hematogenous or lymphatic spread is actually responsible for the metastasis [92].

### 3.4. Radiologically Inserted Gastrostomy

Endoscopically placed percutaneous gastrostomy was very soon followed by the development of radiological techniques for fluoroscopic percutaneous placement [93]. Radiologically inserted gastrostomy implies a procedure where gastrostomy is inserted directly into the stomach under X-ray guidance. Indications and complications are similar to PEG, with the only difference being in the performed technique.

A preliminary CT (computed tomography) scan or ultrasound examination can be performed to rule out an overlapping colon or left hepatic lobe. Sometimes, 100–200 mL of barium sulfate can be orally administered to patients the night before the procedure to sketch the colon. On the day of the procedure, air or CO_2_ gas is insufflated in the stomach via a nasogastric tube to extend the stomach. Afterward, gastric fixation is required and a gastropexy is conducted, which is followed by a gastric puncture. Gastropexy can reduce the spilling of the gastric contents around gastrostomy into the peritoneum and is performed in 93% of cases according to a prospective multicenter survey of the United Kingdom [94,95]. The position in the stomach is confirmed by aspirating air or injecting a contrast medium. After the tube placement, contrast is injected to prove the proper position [96,97].

Minor complications are similar to PEG, except for tube displacement, which happens in 1% of cases. According to Cherian et al., there are no differences in major complications between PEG and RIG at 6.8% and 8.5%, respectively [98]. Moreover, a meta-analysis from Wollman et al. found that the success rate of the tube placement is higher in RIG compared to PEG, while also major complications occurred less often in RIG [99]. Another meta-analysis proved that major complications were more frequent in PEG patients (2.19%) than RIG patients (0.07%), but with no significant statistical difference [100]. Additionally, RIG can be placed in cases of esophageal/oropharyngeal obstruction and since there is no gastroscopy involved, there is no need for antibiotic prophylaxis.

### 3.5. Surgical Gastrostomy

Surgical gastrostomy is nowadays considered a rudimentary technique due to safer and less invasive characteristics of PEG. However, in certain situations, especially in HNC patients, surgical gastrostomy is still a viable option. It is conducted for indications such as when HNC prevents access for gastroscopy, severe strictures of the esophagus or impossibility to set stomach to adjacent to the abdominal wall due to obesity, previous surgery, or hepatosplenomegaly [101].

Surgical gastrostomy can be conducted in two ways: with open laparotomy or by laparoscopy. Laparoscopy is the less invasive and surgically better method due to the better exposure of the stomach since in open laparotomy the incision is commonly very small [102]. Nevertheless, the literature has shown that even laparoscopy gastrostomy placement has significantly more complications compared to PEG [103,104].

### 3.6. Jejunostomy

The jejunostomy feeding tube is a method of nutrition through access to the jejunum. It is used when a gastrostomy tube is not technically possible or contraindicated. It can be administrated endoscopically, radiologically, or by surgery. When placing these feeding tubes, minimally invasive techniques are always preferred [105].

Jejunostomy, as gastrostomy, is preferred for long-term enteral nutrition greater than six weeks. Benefits to this technique compared to the NGT and PEG are lessening the risk of tube malposition, lower risk of aspiration since the gastroesophageal sphincter is not held open by a tube, no nasal discomfort, and pressure injury of the nares [72].

Despite having some advantages, there are also complications including infections, nausea, vomiting, diarrhea, abdominal distension, bowel obstruction, and metabolic abnormalities [106,107]. This method of enteral nutrition is not suitable for patients with bowel obstruction distal to the placement site. Regarding HNC patients, jejunostomy is usually indicated in those patients that have other conditions that prevent using oral or gastric nutrition. When feeding jejunely, a continuous and slow rate is advised due to the loss of the stomach reservoir [51].

## 4. Parenteral Nutrition

Parenteral nutrition represents an intravenously delivered nutritionally balanced synthetic mixture of sterile nutrients [108]. It has to be slowly introduced, starting with 15–20 calories per kg of body weight a day with a maximum of 1000 calories a day. As it is initiated and the caloric levels rise toward the target level, the patient needs to be monitored because of possible drops in potassium, phosphorous, and magnesium levels [109]. Multiple meta-analyses have shown more infectious complications in patients using parenteral nutrition, yet estimated caloric requirements can be more easily accomplished [110]. Moreover, several studies imply that better outcomes occur when at least one part of a patient’s nutrition is via the enteral route [6,111,112]. However, when the intake of food and nutrient absorption becomes almost impossible due to a defective, unavailable, or ruptured gastrointestinal tract, parenteral nutrition is the best and only choice [111]. Hence, the main indication of PN is when there is no other way to meet the required calorie intake. Several other indications are severe catabolic state due to the severity of the patient’s condition (such as sepsis and polytrauma), gastrointestinal malformations (congenital in children or after surgery or cancer), severe vomiting, and diarrhea [113,114,115,116]. Moreover, parenteral nutrition can be used in cases of prolonged chyle leaks that can take place after extensive left neck surgery (the thoracic duct is located nearby) [117]. This type of nutrition is also recommended for terminal care patients [118].

Parenteral nutrition is contraindicated in patients with a functional gastrointestinal tract, patients that do not have access to an intravenous line, and in patients that need therapy for fewer than 5 days if they are not severely malnourished [119]. As soon as the adequate gastrointestinal function is reached, patients are gradually transitioned from parenteral to enteral or oral nutrition to avoid biliary sludge and GI mucosal atrophy. When enteral or oral intake reaches 500 calories a day, parenteral nutrition ought to be decreased, while when more than 60% of caloric needs are achieved via oral or enteral intake, parenteral nutrition should be canceled [119]. Parenteral nutrition is rarely applied to patients with HNC because, in most cases, the lower GI tract is functional. A study performed by Ryu et al. revealed that total parenteral nutrition was USD 11.81 more expensive than certain types of enteral nutrition (feeding via NGT) on a daily basis [120]. Research conducted by Scolapio et al. revealed that many patients would prefer the parenteral type of feeding more than enteral (NGT feeding) if they were unable to eat [118].

When it comes to HNC, patients often deal with morphological malformations, caused both by malignancy and/or treatment [112]. While severe illness such as cancer causes metabolic changes and malnutrition, demanding and extensive surgeries impact and exhaust organism as well [111]. Therefore, intensive follow-up treatment is required in order to enable the patient to recover as soon and as best as possible. When it comes to the nutritional deficits of patients after head and neck surgery, the problem that often occurs is in the upper gastrointestinal area. Some of the surgeries require manipulation and extensive resection in the intraoral space and that can lead to several complications. As previously mentioned, the most common are odynophagia, dysphagia, mucositis, xerostomia, and edema [6]. These complications can induce malnutrition, in which case it is of vital importance to adequately approach this problem. When malnutrition occurs for more than two weeks, there is a risk of developing refeeding syndrome; metabolic changes followed by hypokalemia, hypomagnesemia, and hypophosphatemia; and life-threatening conditions such as cardiac and respiratory distress [121]. To avoid this dangerous state, it is necessary to recognize the nutritional needs of the patient at the time and adjust if enteral feeding is in any way compromised. For parenteral nutrition, an interdisciplinary approach is needed (physicians, nutritionists, and educated staff in the surgical department) in order to reduce potential complications and give the patient the necessary care [119]. In the current literature, there are not many studies regarding this subject. Ackerman et al. evaluated and presented nutrition management for HNC patients, where they discussed and pointed out that prevention of undernutrition is the key to avoiding poor treatment outcomes [6]. On the other hand, there are no specific guidelines when it comes to the nutrition of head and neck cancer patients. While ESPEN brings relevant guidelines on nutrition covering most of the conditions and diseases, on the other hand, when it comes to parenteral nutrition in surgery, ESPEN recommends the formula of 25 kcal/kg ideal body weight [122].

## 5. Conclusions

HNC patients are, due to the anatomical region involved, a highly sensitive group of patients regarding their nutritional management. While oral intake is the most superior way of feeding, that is rarely possible in this particular group. Since they most commonly have a functional gastrointestinal tract, enteral nutrition should be indicated over the parenteral possibility. However, according to the relevant literature, it is still debatable which enteral modality should be administrated. While PEG is starting to be deemed the “golden standard” for these patients, on the other hand, NGT is still considered a highly viable choice. Moreover, even though the literature is very scarce regarding the importance of the pre-surgery preparation and planning of the nutritional route, we believe that it is an integral part of a faster recovery after surgery. Furthermore, none of the relevant sources give any recommendations or guidelines for the nutritional management of these patients. Henceforth, this issue should be given more focus in future studies and an algorithm should be established for an improvement of nutritional support in this particular group of patients.

## Figures and Tables

**Table 1:** Main characteristics and differences between enteral and parenteral nutrition.

Enteral Nutrition	Parenteral Nutrition
Cheaper	Expensive
Lower infection rate	Higher infection rate
Need of monitoring for optimal nutrition	Delivery of optimal nutrition
No gut atrophy	Gut atrophy
Shorter hospitalization rate	Longer hospitalization rate
Fewer complications	More complications

**Table 2:** Enteral nutrition modalities and their characteristics.

Enteral Nutrition Method	Term	Advantages	Disadvantages
Nasogastric tube	Short term	Simple placement; cheap	Discomfort; potential displacement; risk of aspiration
Nasojejunal tube	Short term	Can be used in patients with impaired gastric motility; reduced aspiration risk; cheap	Discomfort; potential displacement; require skilled specialist; no bolus feeding
Gastrostomy			
PEG	Long term	Can be used for a prolonged time; minimally invasive technique	Needs sedation; invasive technique; potential displacement; stoma complications; tumor seeding
RIG	Long term	Can be used for a prolonged time; minimally invasive technique; no sedation needed	Invasive technique; stoma complications; potential displacement due to smaller tubes
surgical	Long term	Can be used for a prolonged time; no tube displacement	Requires surgery under general anesthesia; stoma complications
Jejunostomy	Long term	Can be used for a prolonged time; no risk of aspiration; less discomfort	By-passes the stomach; continuous slow rate of feeding; potential bowel obstruction

Abbreviations: PEG—percutaneous endoscopic gastrostomy; RIG—radiologically inserted gastrostomy.
